# Systematic analysis of the function and prognostic value of RNA binding proteins in Colon Adenocarcinoma

**DOI:** 10.7150/jca.50407

**Published:** 2021-03-05

**Authors:** Xuewei Qi, Zeyu Liu, Qiaoli Zhang, Ming Yang, Yuxiang Wan, Jinchang Huang, Lin Xu

**Affiliations:** 1 Third Affiliated Hospital, Beijing University of Chinese Medicine, Beijing 100029, China.; 2 Institute of Acupuncture and Moxibustion in Cancer Care, Beijing University of Chinese Medicine, Beijing 100029, China.; 3 School of Traditional Chinese Medicine, Beijing University of Chinese Medicine, Beijing 100029, China.

**Keywords:** RNA-binding proteins (RBPs), colon adenocarcinoma (COAD), prognosis analysis, predictive model

## Abstract

**Background:** Abnormal expression of RNA-binding proteins (RBPs) is closely related to tumorigenesis, progression, and prognosis. This study performed systematic bioinformatic analysis of RBPs abnormally expressed in colon adenocarcinoma (COAD) using the Cancer Genome Atlas (TCGA) database to screen prognostic markers and potential therapeutic targets.

**Methods:** First, the gene expression data from COAD samples were used to screen out differentially expressed RBPs for functional enrichment analysis and to visualize interaction relationships. Second, RBPs that were significantly related to prognosis were screened through univariate and multivariate Cox regression analysis to construct a prognostic model. The prediction performance of the prognostic model was evaluated by survival analysis and receiver operating characteristic (ROC) curve analysis. It addition, it was verified in the test cohort. The Human Protein Atlas (HPA) online database was used to verify the expression levels of RBPs in the prognostic model.

**Results:** The study identified 181 differentially expressed RBPs and analyzed their interaction and functional enrichment, which were mainly related to non-coding RNA processing, ribosome biogenesis, RNA metabolic processes, RNA phosphodiester bond hydrolysis, and alternative mRNA splicing. Five RBPs related to prognosis were used to construct a prognostic model, and its predictive ability was verified by the test cohort. ROC curve analysis showed that the prognostic model had good sensitivity and specificity. Independent prognostic analysis showed that risk scores could be used as independent prognostic factors for COAD.

**Conclusion:** This study constructed a reliable prognostic model by analyzing COAD differentially expressed RBPs, facilitating the screening of COAD prognostic markers and therapeutic targets.

## Introduction

Colon cancer is one of the most common and deadly malignant tumors, presenting a serious threat to human life 1. The incidence and morality of colon cancer have been increasing rapidly in recent years 2. Colon adenocarcinoma (COAD) is the most common pathological type of colon cancer 3, with approximately 1.2 million new cases worldwide every year, causing 600,000 deaths 4. Surgical resection is currently the primary treatment for localized COAD. After tumor resection, COAD is prone to relapse and metastasis. The proportion of poor prognosis in COAD patients is 25% to 40% 5, 6. In addition, because the onset of COAD may be occult with no specific symptoms and signs, some patients are usually diagnosed at an advanced stage, which has limited therapeutic outcomes. Despite some progress in COAD diagnosis and treatment in recent years, low survival rates, high recurrence rates, and poor prognoses remain challenging 7. Therefore, actively looking for molecular markers and therapeutic targets to predict the prognosis of COAD patients is of great significance for COAD treatment and improving patient prognoses.

RNA-binding protein (RBP) is an important component in post-transcriptional modification, playing an important role in tumorigenesis and tumor progression 8. Thus far, 1,542 human genes encoding RBPs have been confirmed by experiments, accounting for approximately 7.5% of all protein coding genes 9. RBPs interact with other proteins or RNAs to form ribonucleoprotein complexes, which regulate RNA processing, translation, exportation, and localization, thereby maintaining the stability of the intracellular environment 10. The abnormal function of RBPs in a tumor primarily manifests in two aspects: abnormal RBP expression level, and changes in RBP activities. Related studies have shown that RBP expression in cancer tissues is significantly different from that of adjacent tissues, and is closely related to cancer patient prognosis 11-14. Hence, systematic RBP studies enable further understanding of tumor pathogenesis and therapeutic targets, and thus are important for the identification of therapeutic targets and prognoses. Recently, there have been studies to construct cancer survival models based on RBP expression, as well as to assess the prognosis and screen therapeutic targets 15-17, but no relevant research is available for COAD.

In this study, RPBs related to COAD were obtained from the Cancer Genome Atlas (TCGA) database, and their potential functions were analyzed by identifying RBPs that were differentially expressed between tumor tissues and normal tissues. Subsequently, a prognostic prediction model was constructed to evaluate the prognosis of COAD patients, aiming to find independent prognostic biomarkers, which may better guide clinical COAD treatment.

## Materials and methods

### Data collection and differential expression analysis

In this study, the gene expression data and clinical information from COAD samples were obtained from the TCGA database 18 (https://portal.gdc.cancer.gov; until May 7, 2020). RNA-Seq-FPKM data were downloaded and analyzed from 398 COAD cases and 39 non-tumor tissues. Because the publishing guidelines provided by TCGA were strictly abided by, no ethical approval was required. The Limma package 19 of R software was used for differential expression analysis. The Wilcoxon signed-rank test was used to screen differentially expressed RBPs 9 in the tumor and normal tissues. The cut-off values were < 0.05 for false discovery rate (FDR), and |log2 FC| > 1. Heat maps were generated using pheatmap software.

### Gene ontology and pathway enrichment analysis

To comprehensively analyze the biological functions of these differentially expressed RBPs, the ClusterProfiler package 20 of R software was used for gene ontology (GO) 21 and Kyoto Encyclopedia of Genes and Genomes (KEGG) 22 pathway enrichment analysis. The GO terms included three categories: biological process, cellular component, and molecular function. *P* < 0.05 and *q* < 0.05 were used as statistically significant standards.

### Protein-protein interaction network construction and module selection

To obtain the correlation of differentially expressed RBPs, these differentially expressed proteins were mapped using the Search Tool from the Retrieval of Interacting Genes/Proteins (STRING) 23 (https://string-db.org/) database, followed by utilizing Cytoscape 24 (version 3.6.1) for network visualization. The key modules of the protein-protein interaction (PPI) network were identified by the Molecular Complex Detection (MCODE) plug-in, with both MCODE score and node count number more than five 25. *P* < 0.05 was the threshold for significant difference.

### Prognosis model construction and verification

Univariate Cox regression analysis was performed of RBPs in PPI, and the log-rank test was used to select RBPs related to survival. Subsequently, the entire cohort was divided into training and test cohorts. The survival-related RBPs in the training cohort were analyzed by multivariate Cox regression to construct a prognostic model and calculate a risk score in order to evaluate the prognosis of COAD patients. The formula for calculating the risk score of each sample is as follows:

Risk score = 
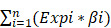


where *β* represents the regression coefficient, and *Exp* represents the gene expression value.

To evaluate and verify the predictive ability of this prognostic model, the patients in the training and test cohorts were divided into low-risk and high-risk groups according to the training cohort's median risk, after which Kaplan-Meier survival analysis was performed to compare the overall survival rates of the two groups of patients. The calculation of *P*-value was performed using the log-rank test. The receiver operating characteristic (ROC) curve was prepared to evaluate the prediction accuracy of the prognosis model. Area under the curve (AUC) >0.6 was considered to be an acceptable model.

Risk score and other clinical variables (e.g., age, gender, stage, and TNM classification) together were subjected to univariate and multivariate Cox regression analyses to determine whether the risk score could be used as an independent prognostic factor. A nomogram was also prepared according to the RBPs in the prognosis model.

### Genetic alteration analysis and verification of expression levels

The cBioPortal 26 (https://www.cbioportal.org/) was used to perform genetic alternation analysis on RBPs in the risk model. The Human Protein Atlas (HPA) 27 online database (http://www.proteinatlas.org/) was used to detect the expression levels of RBPs in the prognostic model.

## Results

### Identification of differentially expressed RBPs in COAD patients

**Figure 1:**
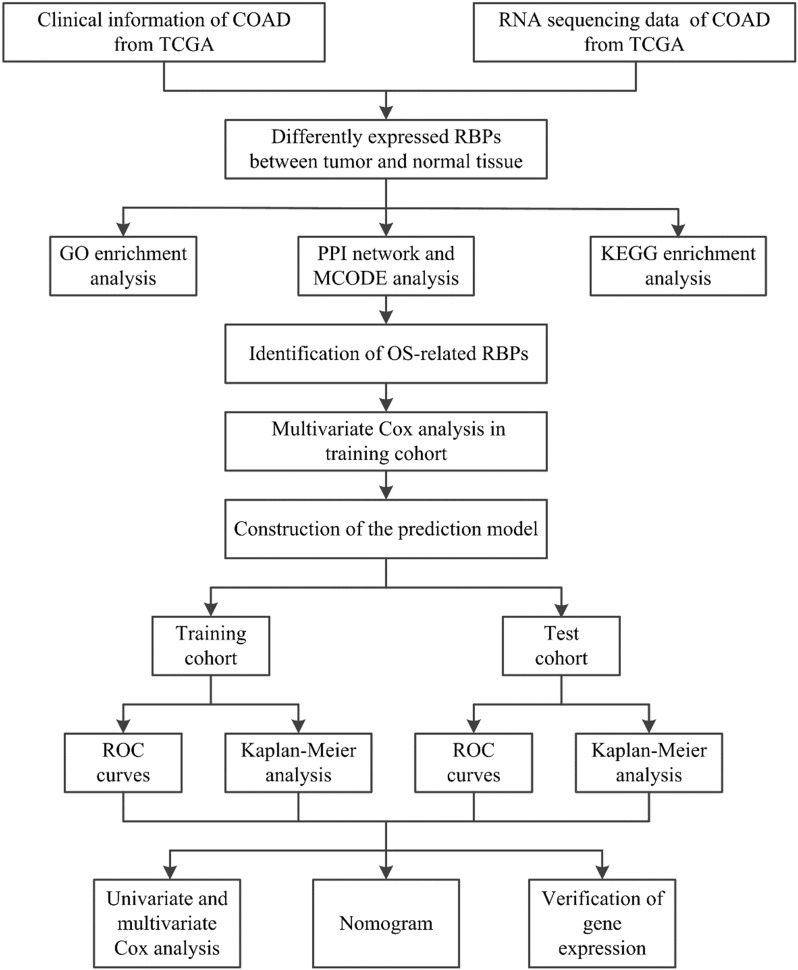
A flow chart of the study design.

**Figure 2:**
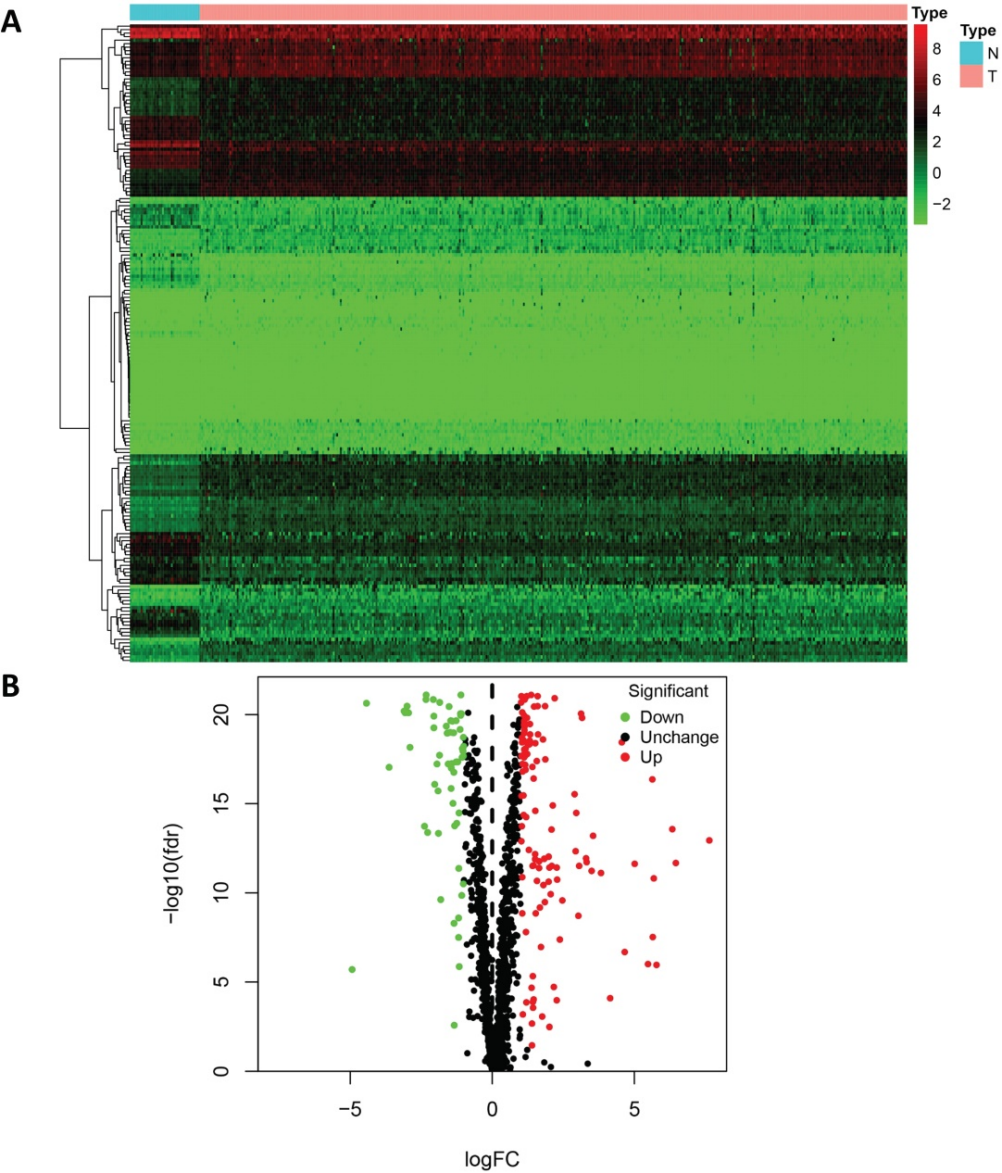
181 differentially expressed RBPs in COAD, including 121 upregulated RBPs and 60 downregulated RBPs. (A) Heat map; (B) Volcano plot.

A flowchart of our study design is shown in Figure 1. Clinical information and gene expression data of 398 COAD samples and 39 non-tumor tissue samples were obtained from the TCGA database, and a total of 1,375 RBPs were collected. After differential expression analysis using the Limma package of R software, 181 differentially expressed RBPs were identified (FDR < 0.05 and |log2 FC| > 1), including 121 upregulated RBPs and 60 downregulated RBPs. The expression distribution of these differentially expressed RBPs is shown in Figure 2. For example, compared to non-tumor tissue, TRIM71 is upregulated and RBFOX3 is downregulated in tumor tissue. Studies have shown that TRIM71 can inhibit the expression of tumor suppressor CDKN1A/p21 and promote the proliferation of tumor cells. TRIM71 is up-regulated in hepatocellular carcinoma patients and is associated with tumor progression and poor prognosis 28.

### Functional enrichment analysis of the differentially expressed RBPs

**Figure 3:**
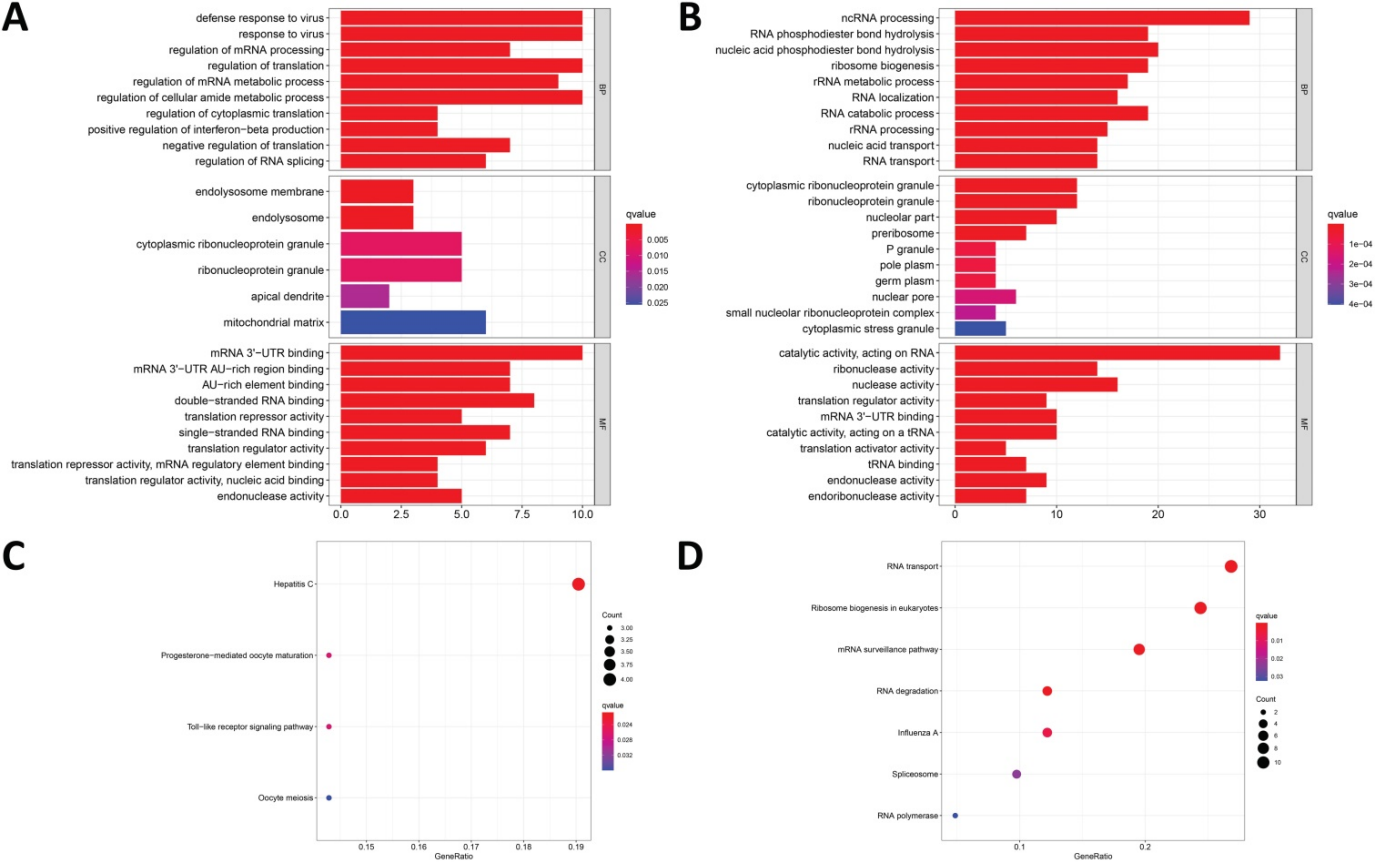
Functional enrichment analysis of the differentially expressed RBPs. GO enrichment analysis (BP/CC/MF) results of (A) downregulated RBPs and (B) upregulated RBPs; KEGG pathway analysis results of (C) downregulated RBPs and (D) upregulated RBPs.

To analyze the biological functions and related signaling pathways of the differentially expressed RBPs, GO and KEGG pathway enrichment analyses were performed. The results of GO enrichment analysis showed that the upregulated differentially expressed RBPs were significantly enriched in non-coding RNA (ncRNA) processing, phosphodiester bond hydrolysis, and ribosome biogenesis. The downregulated differentially expressed RBPs were enriched in the defense response to virus, regulation of mRNA processing, regulation of translation, and regulation of mRNA metabolic processes. In terms of molecular function, the upregulated differentially expressed RBPs showing significant enrichment were involved primarily in catalytic activity acting on RNA, ribonuclease activity, and nuclease activity. The downregulated differentially expressed RBPs were significantly enriched in mRNA 3'-UTR AU-rich region binding, double-stranded RNA binding, and translation repressor activity. The results of the cellular component analysis showed that the upregulated differentially expressed RBPs were mainly enriched in cytoplasmic ribonucleoprotein granules, nucleolar components, and preribosomes, while the downregulated differentially expressed RBPs were mainly enriched in the endolysosomal membrane, cytoplasmic ribonucleoprotein granule, and ribonucleoprotein granule (Figure 3A and 3B).

In addition, the results of the KEGG pathway enrichment analysis showed that the upregulated differentially expressed RBPs were significantly enriched in ribosome biogenesis in eukaryotes, RNA transport, mRNA surveillance pathways, and RNA degradation. The downregulated differentially expressed RBPs were significantly enriched in hepatitis C, progesterone-mediated oocyte maturation, and the toll-like receptor signaling pathway (Figure 3C and 3D).

### PPI network construction and module selection

**Figure 4:**
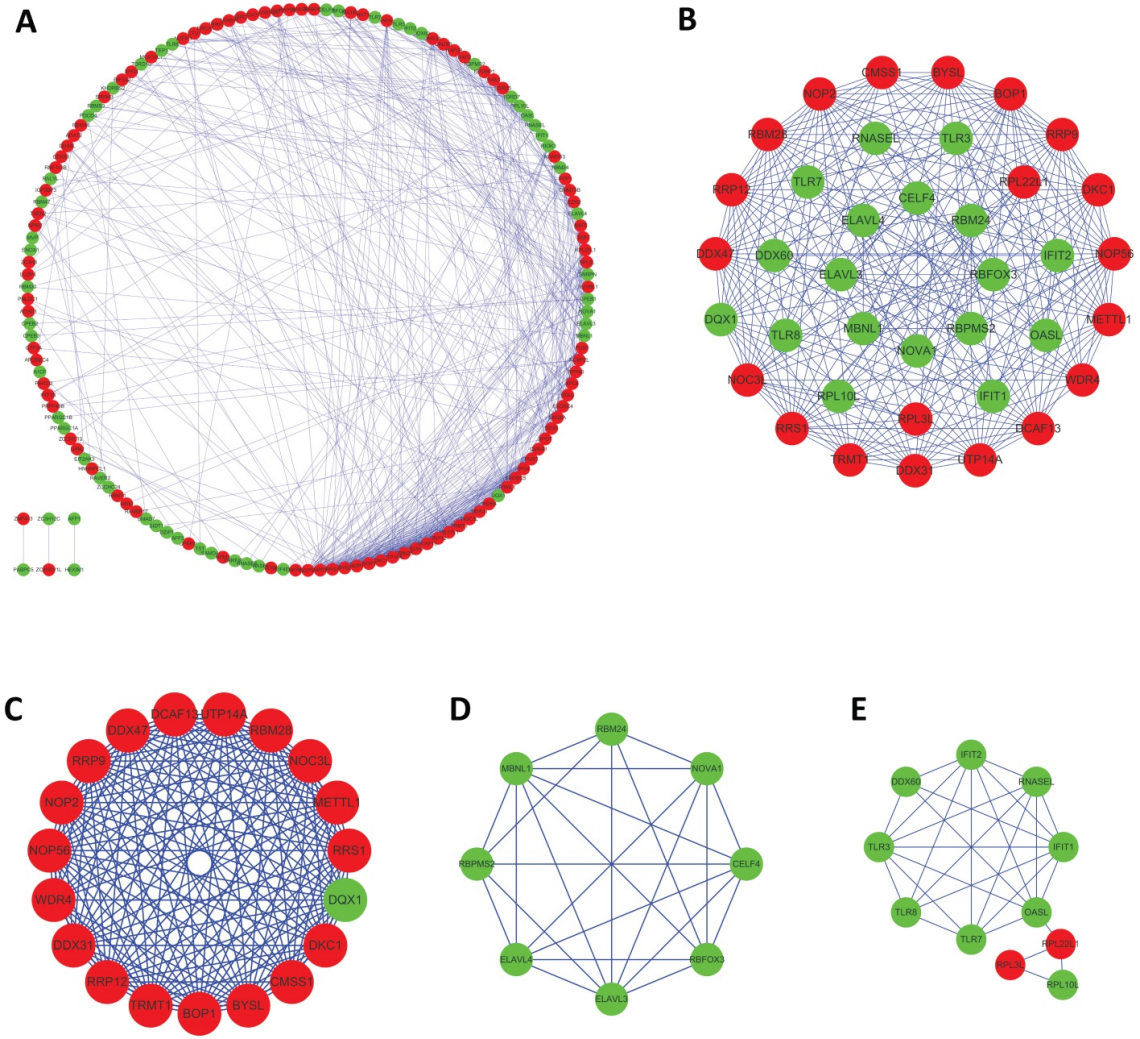
Protein-protein interaction (PPI) network and modules analysis. (A) PPI network of 181 differentially expressed RBPs; (B) critical module from PPI network; (C)critical module 1, (D) critical module 2 and (E)critical module 3 from PPI network. Red nodes: upregulated RBPs; green nodes: downregulated RBPs.

To further examine the interaction between these differentially expressed RBPs, the study utilized a PPI network using the STRING database, which contained 171 nodes and 573 edges, and was visualized using Cytoscape (Figure 4A). The medians of two topological features, degree and betweenness, were selected as the criteria, and seven pivot proteins, namely NOP56, DKC1, DDX31, DDX47, RRS1, METTL1, and PIWIL1 were obtained. Using the MCODE plug-in to process the PPI network, three key modules were selected, including module 1, which contained 19 nodes and 161 edges(Figure 4C); module 2, which contained 8 nodes and 25 edges(Figure 4D); and module 3, which contained 11 nodes and 28 edges (Figure 4D). The results of functional enrichment analysis showed that module 1 was mainly enriched in ncRNA processing, ribosome biogenesis, and rRNA processing; module 2 was mainly enriched in regulation of alternative mRNA splicing, alternative mRNA splicing, and regulation of mRNA splicing; and module 3 was mainly enriched in defense response to virus, response to virus, and type I interferon biosynthetic process.

### Prognosis-related RBP selection

A total of 171 differentially expressed RBPs were identified in PPI. To evaluate the prognostic significance of these RBPs, univariate Cox regression analysis was performed to obtain 15 RBPs related to prognosis: PNLDC1, TDRD5, PTRH1, RBM47, KHDC1L, LUZP4, PPARGC1A, PPARGC1B, CELF4, TERT, POP1, LRRFIP2, EIF4E3, LIN28B, and TDRD7 (Table 1).

### Prognostic model construction and verification

Multivariate Cox regression analysis was performed on the candidate RBPs in the training cohorts to obtain the following five RBPs for the construction of the prognostic model: TDRD5, LUZP4, LRRFIP2, TDRD7, and KHDC1L (Table 2).

**Figure 5:**
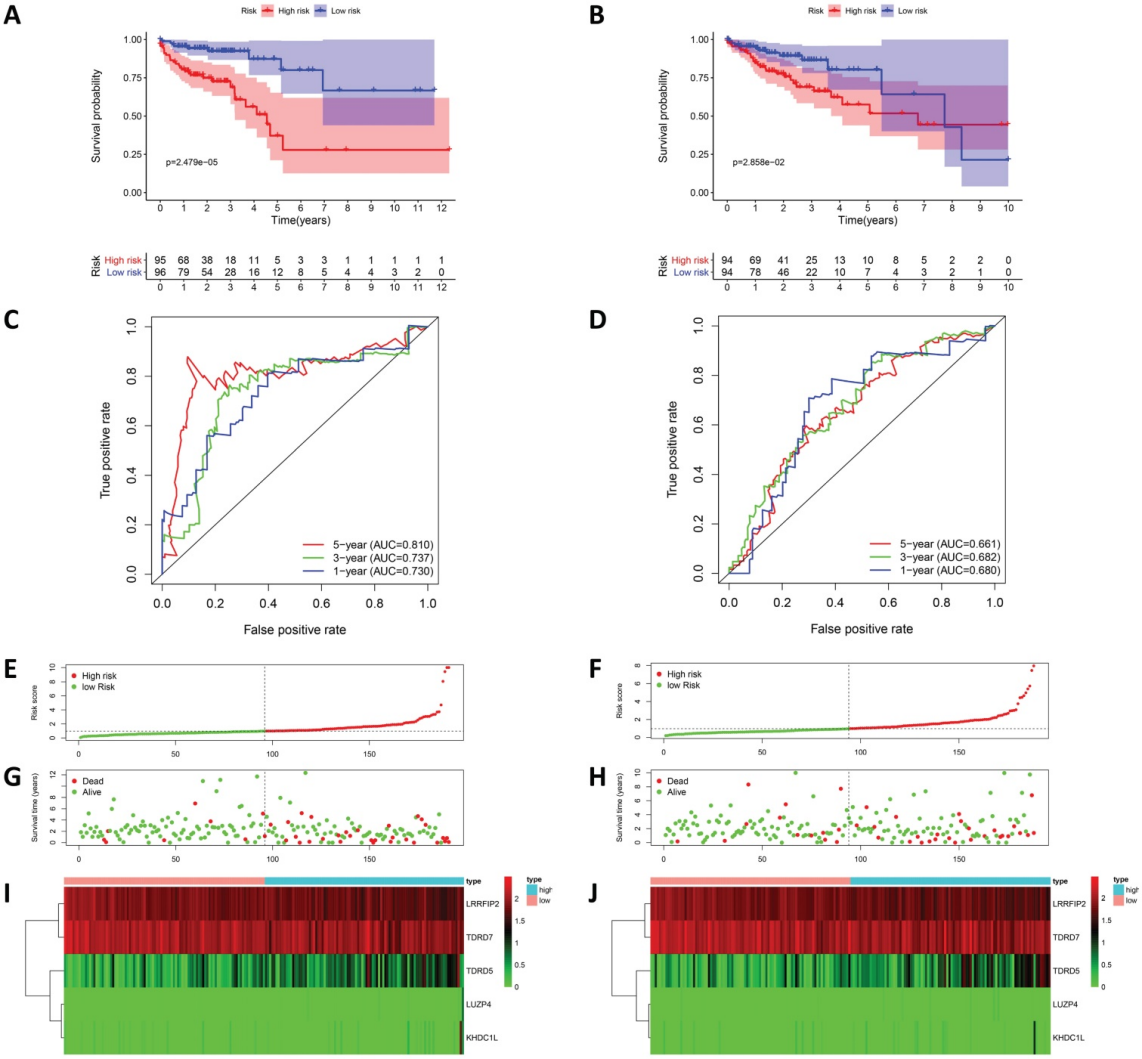
Risk score analysis of prognostic model in TCGA cohort. (A) Kaplan-Meier curves and (C) ROC analysis of the prognostic model in TCGA training cohort; (B) Kaplan-Meier curves and (D) ROC analysis of the prognostic model in TCGA test cohort; (E) risk score curves, (G) survival status and (I) heat map of COAD patients in TCGA training cohort and training cohort.; (F) risk score curves, (H) survival status and (J) heat map of COAD patients in TCGA test cohort.

To evaluate the prognostic model's predicative power, 191 patients in the training cohort were divided into high-risk and low-risk groups for survival analysis based on the median risk score. The results showed that patients in the high-risk group had poorer survival rates than those in the low-risk group (Figure 5A). ROC analysis was used to test the predictive accuracy of our model, and the results showed that our model predicted the prognosis of COAD patients very well. The AUC values under the ROC curves for the one-, three-, and five-year survival rates were0.730, 0.737, and 0.810, respectively (Figure 5C). Figure 5 shows the risk score curves, survival status distributions, and RBPs gene expression heat map of patients in the high and low-risk groups. To further verify the accuracy of this prognostic model, survival analysis and ROC analysis were performed on a test cohort consisting of 188 patients, and the results showed that the survival rates of the high and low-risk groups were significantly different. The AUC values under the ROC curves for the one-, three-, and five-year survival rate were 0.680, 0.682, and 0.661, respectively, suggesting that our prognostic model had good sensitivity and specificity (Figure 5).

**Figure 6:**
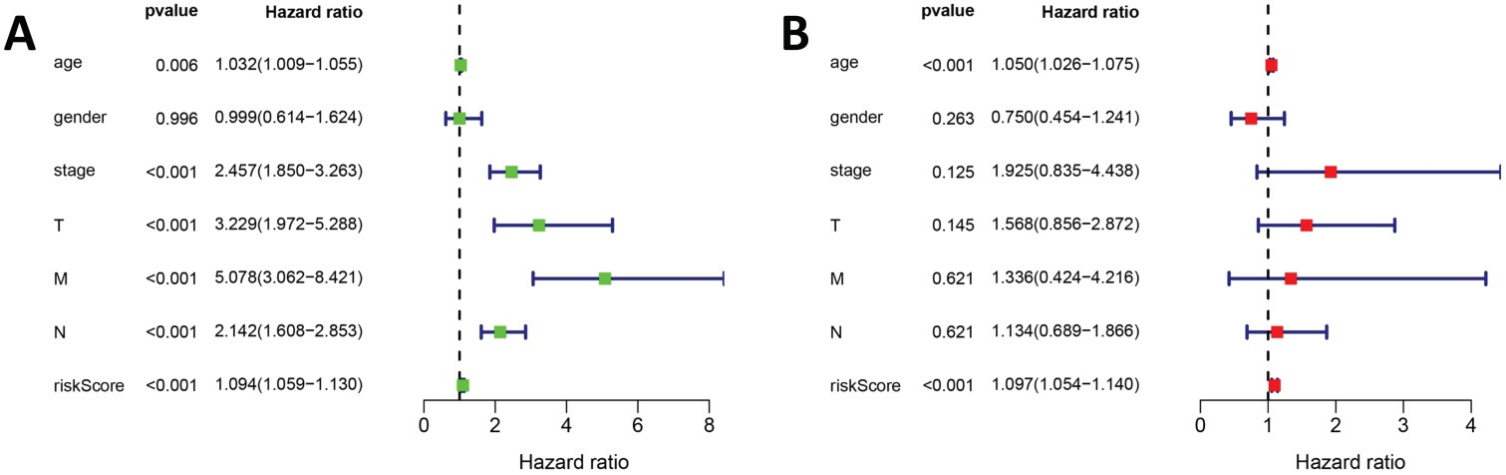
The Cox regression analysis for evaluating the independent prognostic value of the risk score of the prediction model. The (A) univariate and (B) multivariate Cox regression analysis of age, gender, stage, TNM classification and risk score.

In addition, univariate and multivariate Cox regression analyses were used to evaluate the risk scores of our prognostic model and prognostic values of other clinical features. The univariate Cox regression analysis suggested that stage, TNM classification, and risk score were associated with COAD prognosis (Figure 6A). The multivariate Cox regression analysis suggested that risk score may be an independent risk factor (Figure 6B).

### Construction of a nomogram based on RBPs in the prognostic model

**Figure 7:**
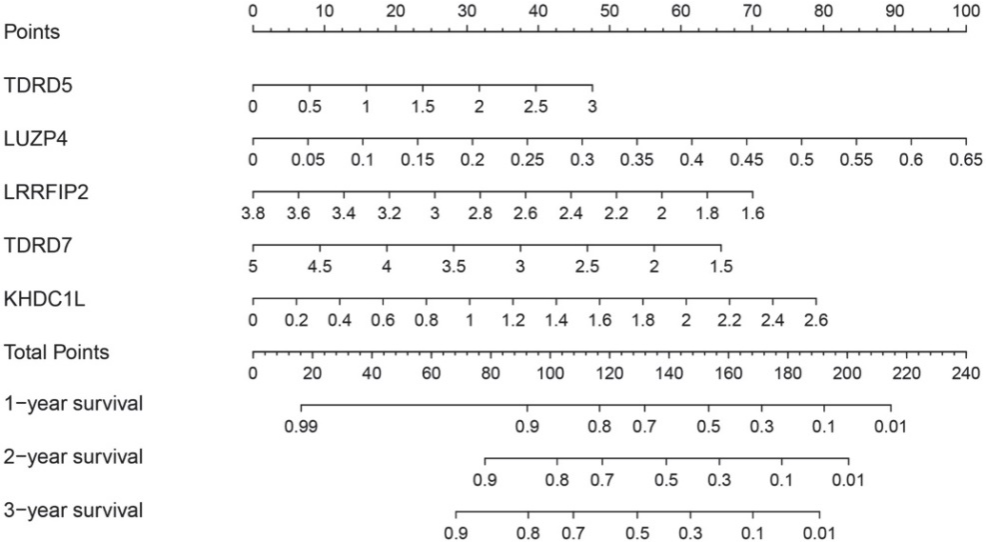
Nomogram of predicting 1-, 2-, and 3-years OS of COAD patients in the TCGA cohort.

To better predict the survival time of COAD patients, a nomogram based on the gene expression of RBPs in the prognosis model was constructed (Figure 7). The expression of each RBP in the prognostic model corresponded to a point. All points were added to calculate the total points of each patient. The one-, two-, and three-year survival rates of the patients were predicted by drawing perpendicular lines between the axis of total points and each prognostic axis.

### Genetic alteration analysis and expression level verification

**Figure 8:**
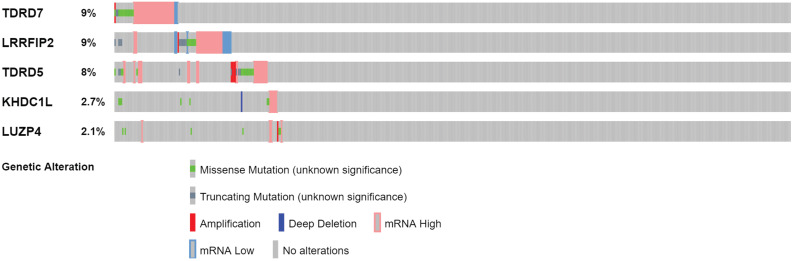
Genetic alteration analysis of 5 prognosis-related RBPs in COAD patients.

**Figure 9:**
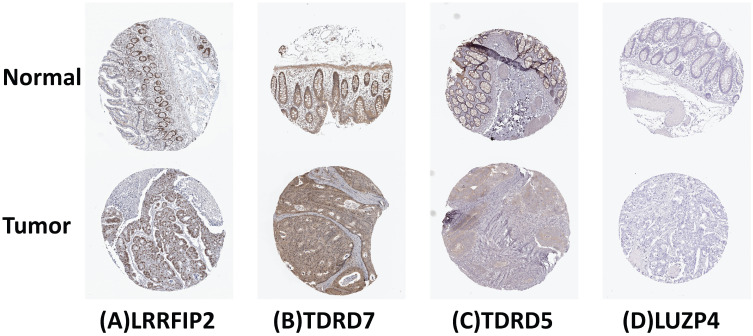
Verification of 4 prognosis-related RBPs expression in COAD and normal colon tissue using the HPA database. (A) LRRFIP2, (B) TDRD7, (C) TDRD5, (D) LUZP4.

The cBioPortal was used to perform genetic alteration analysis on five genes related to prognosis, TDRD5, LUZP4, LRRFIP2, TDRD7, and KHDC1, and the results showed that 130 of 524 COAD samples (25%) had undergone alternation among the listed genes. The alteration was dominated by mRNA upregulation (Figure 8). To verify the differences expression for these five genes, the immunohistochemical results of the five genes expressed in COAD were obtained from the HPA database. LRRFIP2 and TDRD7 expression in COAD tissues were significantly higher than those in the normal colon tissues. In addition, TDRD5 expression was weakly positive in COAD tissues and was negative in normal colon tissues. LUZP4 expression was negative in both normal colon and COAD tissues (Figure 9).

## Discussion

RBPs are an important component of gene expression pathways in eukaryotes and are key regulators of post-transcriptional processes that mediate RNA maturation, transport, localization, and translation 29. The different functions of RBPs in post-transcriptional gene regulation are crucial for cell differentiation and proliferation. Therefore, abnormal expression of RBPs leads to the occurrence of various human diseases, including cancer. RBPs are well recognized to play a key role in tumorigenesis and tumor progression. However, many of their specific roles in cancer biology have not been discovered. Therefore, analysis of the differentially expressed RBP network and related functions is helpful to promote in-depth understanding of their role of tumor biology, possibly revealing new targets for cancer treatment 30. COAD is one of the most common gastrointestinal malignancies, with a low survival rate, high recurrence rate, and poor prognosis. Therefore, screening of prognostic biomarkers and new therapeutic targets has great significance for early therapeutic intervention and prognosis for COAD.

This study analyzed RBPs in 398 COAD and 39 non-tumor tissue samples using TCGA to identify 181 differentially expressed RBPs, including 121 upregulated and 60 downregulated RBPs. The biological functions related to the differentially expressed RBPs were subsequently and systematically analyzed to construct a PPI network. Univariate and multivariate Cox regression analyses, survival analysis, and ROC analysis were used to further explore the biological functions and clinical significance of the differentially expressed RBPs. This study built a prognostic risk model based on five RBPs that were significantly associated with prognosis, and performed cohort verification using the TCGA database. This model can be used to facilitate prognostic analysis and treatment for COAD patients and to develop new biomarkers.

The results of our enrichment analyses of the biological functions of differentially expressed RBPs and related signaling pathways showed that differentially expressed RBPs were enriched on multiple GO terms. In terms of biological process, upregulated differentially expressed RBPs are significant enriched in ncRNA processing, RNA phosphodiester bond hydrolysis, and ribosome biogenesis. Downregulated differentially expressed RBPs are significantly enriched in defense response to virus, regulation of mRNA processing, regulation of translation and regulation of mRNA metabolic process. The ncRNA has been the focus of cancer research 31. The abnormal regulation of lncRNA is related to metastasis and recurrence of many cancers 32. Studies have reported that the expression of ZEB1-AS1 in COAD is significantly upregulated and is related to poor prognosis. The miR-455-3p/PAK2 axis can promote the malignant progression of COAD 33. Ribosomal biogenesis plays an important role in tumorigenesis and tumor progression. Both the upregulation of ribosomal biogenesis and internal dysfunction lead to increased genomic stability and decreased activity of tumor suppressor gene p53, increasing the risk of cancer 34. In terms of molecular function, upregulated differentially expressed RBPs are mainly enriched in scatalytic activity acting on RNA, ribonuclease activity, and nuclease activity. Downregulated differentially expressed RBPs are mainly enriched in mRNA 3'-UTR AU-rich region binding, double-stranded RNA binding, and translation repressor activity. RBPs can bind to various types of RNA and regulate the activity of various enzymes. Ribonuclease can inhibit the proliferation of tumor cells by catalyzing the cleavage of phosphodiester bonds in various single-stranded RNAs 35. In terms of cellular component, upregulated differentially expressed RBPs are mainly enriched in cytoplasmic ribonucleoprotein granule, nucleolar part, and preribosome. Downregulated differentially expressed RBPs are mainly enriched in endolysosome membrane, cytoplasmic ribonucleoprotein granule, and ribonucleoprotein granule. In recent years, mutations in ribosomal protein genes have been found in different types of cancer, such as ribosomal protein S20 (RPS20) gene mutations in colorectal cancer 36. Studies have also shown that ribosomal protein S3 (RPS3) is highly expressed in colon cancer. Knockout of RPS3 can significantly inhibit the proliferation and migration of colon cancer Caco-2 cells, up-regulate the expression of p53 protein, and increase tumor cell apoptosis 37. The KEGG pathway enrichment analysis showed that abnormally expressed RBPs regulated tumorigenesis and COAD progression by affecting ribosome biogenesis in eukaryotes, as well as RNA transport, mRNA surveillance pathways, and RNA degradation. In a previous study that included expression of RBPs in 16 different types of cancers 38, the results showed that RBPs with functions in RNA splicing, translation, transcription termination, RNA localization and transport, RNA surveillance and degradation, RNA modification, ribosome, transfer RNA (tRNA), and other functions were significantly upregulated in COAD tissues, which were consistent with our findings.

In addition, a PPI network of differentially expressed RBPs was constructed to obtain seven hub proteins, including NOP56, DKC1, DDX31, DDX47, RRS1, METTL1, and PIWIL1. Enhanced ribosomal biogenesis and increased protein synthesis are important features of cancer cell proliferation 39. NOP56 is a core protein member of box C/D small nucleolar RNPs (snoRNPs) and plays an important role in ribosome biosynthesis 40. DKC1 encodes dyskerin protein and is involved in rRNA processing, folding, and modification. Multiple studies have shown that DKC1 is upregulated in colorectal cancer, prostate cancer, breast cancer, and other cancers, indicating that DKC1 upregulation may be a common feature of invasive cancer 42, 42. DDX31 is a nucleolar protein, and DDX31 overexpression is related to p53 mutation and estimated glomerular filtration rate (eGFR), which promotes the invasion and migration of bladder cancer 43. DDX31 interacts with and co-localizes with the NPM1 protein in the nucleolus of kidney cancer cells, regulating the p53 pathway and rRNA gene transcription, thereby playing a key role in tumorigenesis and progression of kidney cancer 44. DEAD-box RNA helicases also play important roles in ribosome biogenesis, RNA processing and folding, RNP remodeling, RNA nuclear export, the regulation of RNA translation and transcription, and other processes that are closely related to tumorigenesis. DDX47 is an important helicase that is mainly involved in pre-rRNA processing 45. The main function of RRS1 is to participate in ribosome biogenesis. A previous study has shown that RRS1 expression in colorectal cancer is higher than in the tumor-adjacent normal tissues. Downregulation of RRS1 induces G2/M cell cycle arrest, apoptosis, and angiogenesis, thereby inhibiting the proliferation of colorectal cancer cells 46. In addition, RRS1 overexpression is related to the tumorigenesis and progression of several tumor types, such as gastric cancer, hepatocellular carcinoma, and cervical cancer 47-49. RNA modification is related to the tumorigenesis of various cancers, and the METTL family is a key modifier of tRNA and rRNA. METTL1 mainly regulates the modification of N7-methylguanosine (m7G) and plays an important role in the progression of tumors, such as colon cancer, liver cancer, and lung cancer 50-52. PIWIL1 is an important member of the Argonaute protein family that is closely related to the biological behaviors of tumor cell proliferation, apoptosis, adhesion, metastasis, and chemotherapy resistance 53. Studies have shown that the expression of PIWIL1 in colorectal cancer is significantly higher than that of the tumor-adjacent tissues, and that its expression is closely related to the degree of tumor differentiation, depth of tumor invasion, and TNM stage, thereby promoting the growth, proliferation, and invasion of colorectal cancer 54,55. By analyzing the main modules in the PPI network, we found that these modules were primarily related to ncRNA processing, ribosome biogenesis, regulation of alternative mRNA splicing, and defense response to virus. RBPs-mediated post-transcriptional regulation is one of the important regulatory mechanisms of lncRNA, which mainly regulates lncRNA stability, transport and localization, and plays an important role in the occurrence and development of cancer 56. For example, heterogeneous nuclear ribonucleoprotein K (hnRNPK), a RBP that plays a role in the nuclear accumulation of lncRNAs, is upregulated in colorectal cancer, gastric cancer and other cancers, and is associated with poor prognosis^57^,^58^. RBP can participate in the biogenesis of ribosomes, and the abnormality of ribosomal biogenesis is closely related to the occurrence and development of cancers 59. RBPs regulate gene expression through post-transcriptional regulation, such as alternative mRNA splicing and microRNA processing. Abnormal mRNA splicing is a common driving factor in the occurrence and development of cancer, affecting the phenotype of cancer cells, including proliferation, apoptosis, invasion and transfer 60. In colorectal cancer, SRSF3 interacts with the small splice-regulating protein SRSP, mediating selective splicing of SP4 to produce cancerous SP4 subtypes, which leads to tumor occurrence and metastasis 61.

Subsequently, 15 RBPs related to prognosis were obtained based on univariate Cox regression, and five RBPs (TDRD5, LUZP4, LRRFIP2, TDRD7, and KHDC1L) obtained from the multivariate Cox regression analysis using the TCGA training cohort were used for the construction of a prognostic risk model. TDRD5 could be combined with PIWI-interacting RNA (piRNA) precursors, and plays a key role in piRNA biogenesis 62. A previous study has shown that TDRD5 expression is upregulated in hepatocellular carcinoma, which has value for determining the prognosis of hepatocellular carcinoma 63. LUZP4 is an mRNA export adaptor that is highly silent in normal tissues other than testes, and is frequently activated in cancers, such as lung cancers, ovarian cancer, melanoma, and multiple myeloma 64,65. Another study has shown that LRRFIP2 is involved in the selective cleavage of colon cancer and prostate cancer 66. In addition, LRRFIP2 contains a serin-rich domain that interacts with MyD88 protein, which plays a key role in toll-like receptor 4-mediated signal transduction, and regulates the activity of downstream nuclear factor kappa-light-chain-enhancer of activated B cells (NF-κB) activity 67. Chromatin dynamics regulate a variety of cell functions, and the destruction of chromatin's homeostasis leads to tumorigenesis and tumor progression. A study has shown that TDRD7 may be used as a histone-binding protein and is crucial in regulating chromatin homeostasis 68. Currently, few studies on the role of KHDC1L in tumors are available.

Furthermore, the reliability and stability of our model was also analyzed. The results showed that the model accurately distinguished between patients with different prognoses. The ROC curve analysis also showed that our prognostic risk model has good diagnostic capability. The survival analysis and ROC curve analysis were verified in a TCGA test cohort, and the results also support the above conclusion. The univariate and multivariate Cox regression analyses showed that our model independently predicted the prognosis of COAD patients. Subsequently, a nomogram based on our model was constructed to predict the prognosis of COAD patients more intuitively. The HPA database was used to verify the expression of five hub RBPs at the immunohistochemical level. The results showed that the expression of LRRFIP2 and TDRD7 in COAD tissues was significantly higher than in the normal colon tissues, suggesting that LRRFIP2 and TDRD7 may have potential carcinogenic risk, which was consistent with our previous findings. In addition, expression of LUZP4 was not detected in normal colon or COAD tissues, which may be related to the limitations of data included in the databases. In summary, our prognostic prediction model was relatively reliable and could be used to identify COAD patients with poor prognoses, facilitating the early intervention and treatment of COAD patients.

In general, the prognostic model constructed by this study based on five prognosis-related RBPs predicted the prognosis of COAD patients well and helped physicians to make clinical decisions. However, this study still has certain limitations. First, as our prognostic model used data obtained from the TCGA database, our findings will need to be verified in a larger clinical patient cohort in the future. And since TCGA data are from the United States, the conclusions of this study need to be verified in data from other countries and regions to determine whether the results can reflect the situation in other groups. Second, for the specific role of the selected RBPs in COAD, *in vitro* and animal experiments will be needed in the future to reveal their internal mechanisms.

## Conclusion

This study systemically analyzed the key role and prognostic value of RBPs in COAD by performing univariate and multivariate Cox regression analyses on RBPs that were differentially expressed in tumor and normal tissues. The study ultimately screened five RBPs that were significantly related to COAD prognosis in order to construct a prognostic model. Survival analysis and independent prognosis analysis confirmed that our prognostic model could be used as independent predictor of COAD prognosis. This study helped to further explore the mechanisms of COAD tumorigenesis and progression, and guide the selection of clinical prognostic molecular markers and therapeutic targets for COAD patients.

## Figures and Tables

**Table 1:** 15 RBPs identified by univariate Cox regression analysis

RBP name	Hazard ratio	Lower 95% CI	Upper 95% CI	*P*-value
PNLDC1	1.5865	1.0941	2.3007	0.0149
TDRD5	1.5915	1.0526	2.4063	0.0275
PTRH1	8.2750	1.2506	54.7549	0.0284
RBM47	0.5290	0.3271	0.8556	0.0094
KHDC1L	3.1476	1.6515	5.9989	0.0005
LUZP4	433.7976	18.7845	10017.8796	0.0002
PPARGC1A	0.5544	0.3653	0.8414	0.0056
PPARGC1B	0.5061	0.2719	0.9421	0.0317
CELF4	8.4467	2.4421	29.2156	0.0008
TERT	1.5662	1.0489	2.3388	0.0283
POP1	0.5661	0.3232	0.9914	0.0466
LRRFIP2	0.2851	0.1347	0.6038	0.0010
EIF4E3	0.6406	0.4133	0.9930	0.0465
LIN28B	2.4368	1.3230	4.4882	0.0043
TDRD7	0.4891	0.2767	0.8646	0.0139

**Table 2:** Five prognosis-related RBPs identified by multivariate Cox regression analysis

RBP name	Coef	Hazard ratio	Lower 95% CI	Upper 95% CI	*P*-value
TDRD5	0.9266	2.5260	1.2790	4.9888	0.0076
LUZP4	4.6049	99.9749	3.6527	2736.3292	0.0064
LRRFIP2	-1.3517	0.2588	0.0807	0.8294	0.0229
TDRD7	-0.6481	0.5230	0.2226	1.2290	0.1370
KHDC1L	0.8040	2.2345	0.9579	5.2124	0.0628
